# Interaction of RIPK1 and A20 modulates MAPK signaling in murine acetaminophen toxicity

**DOI:** 10.1016/j.jbc.2021.100300

**Published:** 2021-01-16

**Authors:** Andrea Iorga, Katherine Donovan, Layla Shojaie, Heather Johnson, Janet Kwok, Jo Suda, Brian T. Lee, Mariam Aghajan, Ling Shao, Zhang-Xu Liu, Lily Dara

**Affiliations:** 1 Division of Gastrointestinal and Liver Diseases, Department of Medicine, Keck School of Medicine of University of Southern California, Los Angeles, California, USA; 2 USC Research Center for Liver Disease, Keck School of Medicine of University of Southern California, Los Angeles, California, USA; 3 Cedar Sinai Medical Center, Los Angeles, California, USA; 4 IONIS Pharmaceutical, Carlsbad, California, USA

**Keywords:** TNFAIP3, necrosis, cell death, necroptosis, RIPK3, RIPK1, hepatotoxicity, drug induced liver injury, drug action, receptor interacting protein, A20^HepCKO^, targeted hepatocyte A20 knockout, AAC, asparagine, ALT, alanine aminotransferase, APAP, acetaminophen, ASK1, apoptosis signal-regulating kinase 1, ASO, antisense oligonucleotide, Co-IP, Coimmunoprecipitation, DMSO, Dimethyl sulfoxide, DYKDDDK Tag, Flag Tag, GAC, aspartic acid, GAPDH, glyceraldehyde-3-phosphate dehydrogenase, GSH, glutathione, JNK, c-Jun N-terminal kinase, LPS, lipopolysaccharide, MAPK, mitogen-activated protein kinases, MKK4, MAP2 kinase mitogen-activated protein kinase 4, MLKL, mixed lineage kinase domain like pseudokinase, NAPQI, N-acetyl-p-benzoquinone imine, Nec-1, necrostatin-1, NFkB, nuclear factor kB, PHB1, prohibitin-1, PMH, primary mouse hepatocyte, RCD, regulated cell death, RHIM, RIP homotypic interaction motif, RIPK1/3, receptor interacting protein kinase-1/3, RIPK1^D138N^, kinase dead RIPK1 knock-in, RIPK1^HepCKO^, targeted hepatocyte RIPK1 knockout, ROS, reactive oxygen species, siRNA, small interfering RNA, TBP, TATA box binding protein, TNF, tumor necrosis factor, TNFAIP3, TNF-induced protein 3, also known as A20

## Abstract

Acetaminophen (APAP)-induced liver necrosis is a form of regulated cell death (RCD) in which APAP activates the mitogen-activated protein kinases (MAPKs) and specifically the c-Jun-N-terminal kinase (JNK) pathway, leading to necrotic cell death. Previously, we have shown that receptor interacting protein kinase-1 (RIPK1) knockdown is also protective against APAP RCD upstream of JNK. However, whether the kinase or platform function of RIPK1 is involved in APAP RCD is not known. To answer this question, we used genetic mouse models of targeted hepatocyte RIPK1 knockout (RIPK1^HepCKO^) or kinase dead knock-in (RIPK1^D138N^) and adult hepatocyte specific knockout of the cytoprotective protein A20 (A20^HepCKO^), known to interact with RIPK1, to study its potential involvement in MAPK signaling. We observed no difference in injury between WT and RIPK^1D138N^ mice post APAP. However, RIPK1^HepCKO^ was protective. We found that RIPK1^HepCKO^ mice had attenuated pJNK activation, while A20 was simultaneously upregulated. Conversely, A20^HepCKO^ markedly worsened liver injury from APAP. Mechanistically, we observed a significant upregulation of apoptosis signal-regulating kinase 1 (ASK1) and increased JNK activation in A20^HepCKO^ mice compared with littermate controls. We also demonstrated that A20 coimmunoprecipitated (co-IP) with both RIPK1 and ASK1, and that in the presence of RIPK1, there was less A20-ASK1 association than in its absence. We conclude that the kinase-independent platform function of RIPK1 is involved in APAP toxicity. Adult RIPK1^HepCKO^ mice are protected against APAP by upregulating A20 and attenuating JNK signaling through ASK1, conversely, A20^HepCKO^ worsens injury from APAP.

Acetaminophen (APAP) remains the leading cause of acute liver failure in the United States (1). APAP's reactive metabolite, NAPQI, is inactivated by glutathione (GSH). Upon GSH depletion, NAPQI covalently binds to intracellular proteins causing organelle stress (2). NAPQI targeting of mitochondria results in ROS production, which eventually leads to the collapse of the mitochondrial membrane potential and necrosis (3). APAP-induced hepatocyte necrosis is a form of regulated cell death (RCD), as interfering with multiple signaling proteins has been shown to dampen or prevent APAP hepatotoxicity (4, 5). The mediator of APAP-induced cell death is c-Jun-N-terminal kinase (JNK) (6). It is well known that interfering with the mitogen-activated protein kinase (MAPK) cascade and preventing sustained JNK activation by inhibiting/silencing JNK, MKK4, or ASK1 results in protection from APAP (6, 7, 8, 9, 10, 11). The best described form of necrotic RCD to date is necroptosis, which involves the receptor interacting protein kinases 1 and 3 (RIPK1 and RIPK3) (12). RIPK1 and RIPK3 are multifunctional proteins and key regulators of death and survival. Although RIPK1 was initially discovered as a tumor necrosis factor (TNF) receptor interacting protein and an activator of nuclear factor κB (NFκB) and MAPK signaling, in recent years it has garnered much attention as the initiator of the necroptosis pathway with RIPK3 (13). Abrogation of RIPK1 kinase function *via* genetic mutations or the use of inhibitors prevents certain types of RIPK1-mediated cell death such as apoptosis and necroptosis (13). The interaction of RIPK1 and RIPK3 *via* their shared RIP homotypic interaction motif (RHIM) domain leads to the oligomerization and activation of RIPK3, which recruits and phospho-activates the pseudokinase mixed lineage kinase domain like (MLKL) (14). In the final step in the necroptosis pathway, phospho-MLKL oligomerizes and translocates to the cell membrane where it forms pores in the lipid bilayer resulting in cell membrane permeabilization (15). As MLKL is the effector of the necroptosis pathway, this cell death mode is MLKL-dependent. Initial experiments using the RIPK1 inhibitor necrostatin-1 (Nec-1) have demonstrated that Nec-1 is protective against APAP-induced RCD, leading some to conclude that the hepatocyte death in APAP is a form of necroptosis (16, 17, 18, 19). We observed protection from APAP-induced cell death using antisense oligonucleotide (ASO) knockdown of RIPK1 (20). In support of this, others have confirmed RIPK1's importance in mediating APAP toxicity using siRNA knockdown of RIPK1 (21). However, we subsequently provided definitive evidence that APAP toxicity proceeds through a necrotic but nonnecroptotic form of RCD by demonstrating that both RIPK3−/− mice and MLKL−/− mice are susceptible to APAP toxicity. Therefore, APAP toxicity proceeds through a necrotic but nonnecroptotic form of RCD (20, 22). Despite the lack of a role for RIPK3 and MLKL, antisense-mediated knockdown of RIPK1 protected mice against APAP upstream of JNK, suggesting a necroptosis-independent role for this multifunctional protein (20, 21, 22).

TNF-induced protein 3 (TNFAIP3), or A20 is a ubiquitin-editing and NFκB responsive protein with prosurvival cytoprotective properties, which is known to interact with RIPK1 in the context of TNF signaling (23, 24). Embryonic liver parenchymal cell A20 knockout mice are viable and healthy at birth but develop moderate chronic liver inflammation by 25 weeks of age (25). These mice have no phenotype under basal conditions but display increased liver injury and sustained JNK activation in response to lipopolysaccharides (LPS) (25). However, the role of A20 in hepatocyte necrosis has not been examined, and its relation to RIPK1 in the liver is largely unknown. A20 has also been shown to affect MAPK signaling through interaction with apoptosis signal-regulated kinase-1 (ASK1), resulting in inhibition of JNK activation (26, 27, 28).

Based on our prior work suggesting a role for RIPK1 in APAP hepatotoxicity, we set out to determine whether the kinase-dependent or -independent function of RIPK1 participates in the APAP cell death pathway. To address this question, we examined the kinase inactive RIPK1 (RIPK1^D138N^) transgenic mice (29) and the adult hepatocyte-specific RIPK1 conditional knockout (RIPK1^HepCKO^) mice in APAP-induced liver injury. The hepatocyte-specific knockout mice were significantly protected against APAP necrosis, while the kinase dead RIPK1^D138N^ mice remained sensitive. Mechanistically, we report for the first time that liver-specific knockout of RIPK1 results in increased A20 protein levels. Conversely, hepatocyte conditional knockout of A20 (A20^HepCKO^) promoted sustained JNK activation through increased ASK1 and A20^HepCKO^ mice had more severe liver injury from APAP.

## Results

### RIPK1 kinase-independent function participates in APAP necrosis

**Figure 1:**
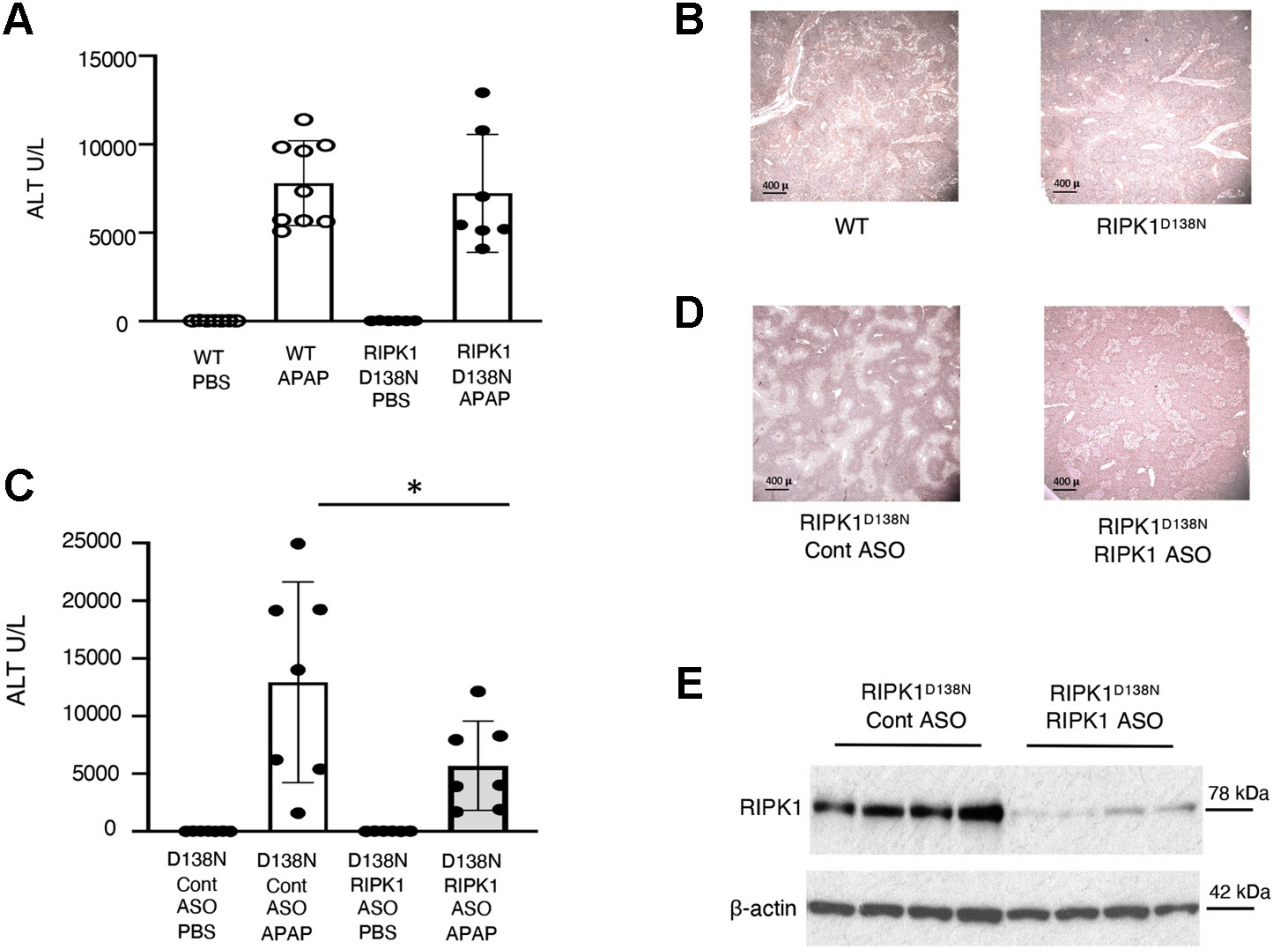
**Kinase dead RIPK1**^**D138N**^**mice are not protected from APAP toxicity *in vivo* while RIPK1 knockdown in RIPK1**^**D138N**^**mice is protective**. *A* and *B*, WT and RIPK1^D138N^ mice were fasted overnight and treated with APAP (300 mg/kg) and euthanized at 24 h. *A*, serum ALT (U/L). *B*, representative histology H&E (4x). *C* and *D*, RIPK1^D138N^ mice were treated with RIPK1 or Control ASO five times, subsequently injected with APAP (300 mg/kg) after overnight fast and euthanized at 24 h. *C*, serum ALT (U/L). *D*, representative histology H&E (4x). *E*, WB of RIPK1 and loading control in RIPK1^D138N^ mice treated with RIPK1 or Control ASO. ∗*p* value ≤ 0.05 RIPK1 *versus* Control ASO treated mice (N = 11–12/group). Results of at least three independent experiments. ALT, alanine aminotransferase; ASO, antisense oligonucleotide; GAPDH, glyceraldehyde 3-phosphate dehydrogenase; RIPK1, receptor interacting protein kinase 1; RIPK1^D138N^, RIPK1 kinase dead knock-in mice; WT, wild type.

We have previously shown that knockdown of RIPK1 in mice was protective against APAP toxicity (20). RIPK1 has both a platform and a kinase function (13). In order to determine which is important in the APAP necrosis pathway, we compared WT mice with age and substrain-matched RIPK1 kinase dead knock-in animals (RIPK1^D138N^). We observed no difference in toxicity following 300 mg/kg injection of APAP after 24 h (Fig. 1, *A* and *B*). However, antisense-mediated knockdown of RIPK1 protein in the RIPK1^D138N^ mice resulted in a significant reduction in ALT, as well as reduced necrosis on histology (Fig. 1, *C–E*). Therefore, the kinase function of RIPK1 is dispensable for APAP induced hepatocyte death while its platform function is required.

Nec-1 has been shown to prevent primary mouse hepatocyte (PMH) death from APAP. However, it is known that Nec-1 has many off-target effects (30). We therefore investigated the effect of a more specific RIPK1 inhibitor, Nec-1s, in this *in vitro* model of cell death. We used PMHs isolated from RIPK1^D138N^ mice that are already lacking kinase function. Nec-1, but not Nec-1s, protected RIPK1^D138N^ PMHs against APAP, confirming the off-target effect for Nec-1 (Fig. S1). We next explored the effect of Nec-1s pretreatment with APAP *in vivo* and found no protection in either WT mice (Fig. S2, *A* and *B*) or RIPK1^D138N^ mice (Fig. S2, *C* and *D*), confirming that RIPK1 kinase function is not involved in APAP toxicity.

**Figure 2:**
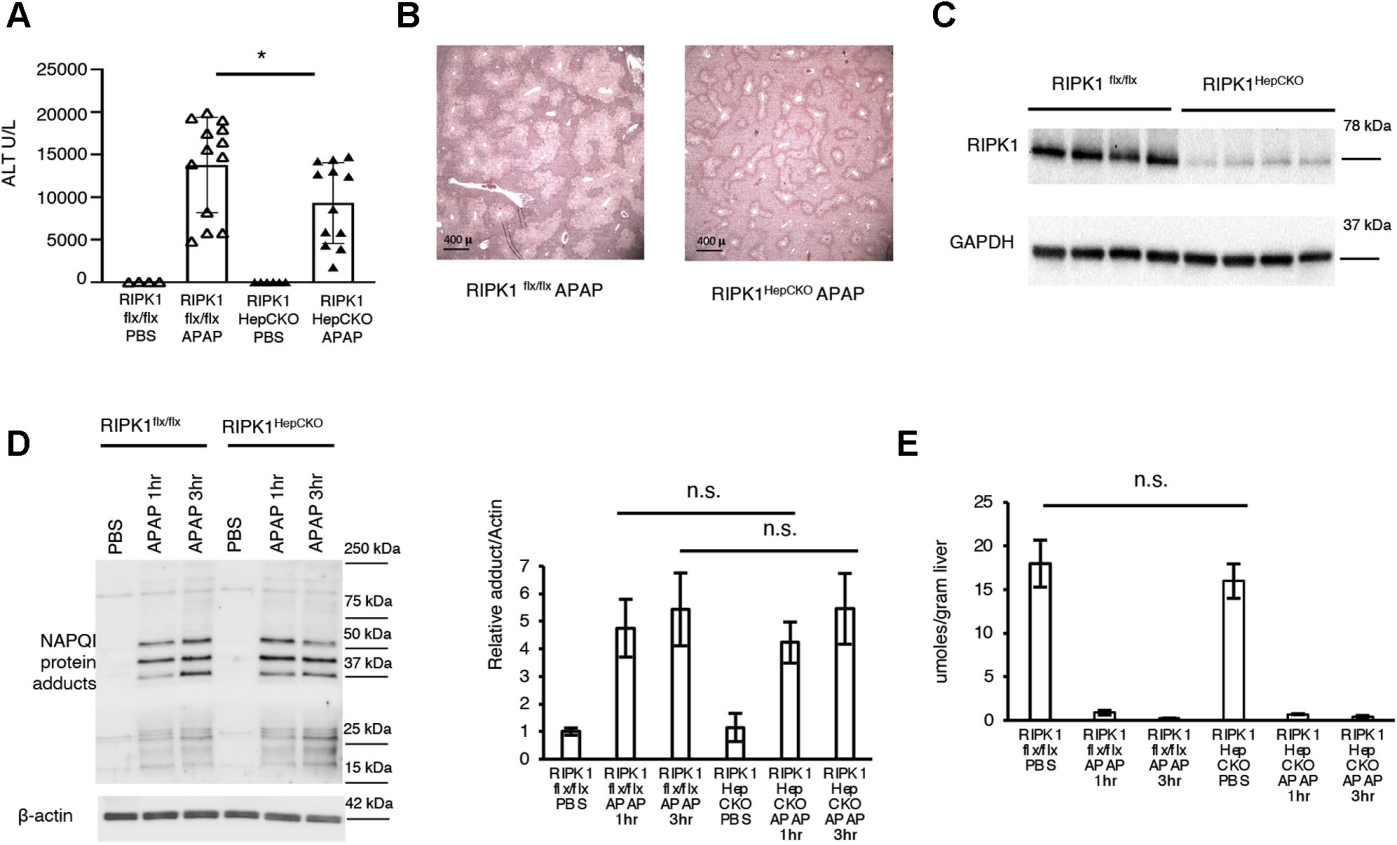
**Hepatocyte-specific RIPK1 knockout (RIPK1**^**HepCKO**^**) protects against APAP toxicity *in vivo***. RIPK1^flx/flx^ mice were treated with AAV8-TBG-iCRE or AAV8-TBG-eGFP for hepatocyte-specific knockout (RIPK1^HepCKO^). Ten days later mice were treated with PBS or APAP (300 mg/kg) after overnight fast and euthanized at 1 h, 3 h, (for GSH and NAPQI adducts) or 24 h (for ALT). *A*, serum ALT U/L at 24 h (N = 12–13/group). *B*, representative histology H&E (4x). *C*, WB of RIPK1 and loading control showing protein knockout. *D*, representative WB of NAPQI protein adducts, densitometry, and loading control, 1 h and 3 h (N = 6/group). *E*, GSH at 1 h and 3 h using a colorimetric recycling assay expressed as μmoles/gram liver (N = 3/group). ∗*p* value ≤0.05 RIPK1^HepCKO^*versus* RIPK1-flx/flx mice ∗∗*p* value ≤0.01 RIPK1^HepCKO^*versus* RIPK1-flx/flx. Results of at least three independent experiments. ALT, alanine aminotransferase; APAP, acetaminophen; Flx, Floxed; GFP, green fluorescent protein; GAPDH, glyceraldehyde 3-phosphate dehydrogenase; NAPQI, N-acetyl-p-benzoquinone imine; N.S., not significant; PBS, phosphate buffered saline; RIPK1, receptor interacting protein kinase 1.

In order to demonstrate that indeed it is the RIPK1 platform function that is important in hepatocytes during APAP overdose, we knocked out RIPK1 in adult RIPK1^flx/flx^ mice using adeno-associated virus-8 (AAV8) coupled to a hepatocyte-specific promoter, tyrosine-binding globulin (TBG), and the causes recombinase (CRE) recombinase. We treated floxed littermate controls with the same dose of AAV8-TBG-eGFP. After 10 days to ensure that RIPK1 hepatocyte conditional knockout (RIPK1^HepCKO^) occurred, we observed no basal inflammation or ALT abnormalities in these mice. Subsequently, mice were fasted overnight and treated with APAP (300 mg/kg). We observed a significant reduction in ALT levels, as well as improved liver histology, in the RIPK1^HepCKO^ compared with floxed littermates (Fig. 2, *A–C*). RIPK1^HepCKO^ did not alter APAP metabolism, as these mice showed the same level of NAPQI adduct formation and GSH depletion as littermate controls (Fig. 2, *D* and *E*).

Unlike RIPK1, which is known to be expressed in hepatocytes, there is much controversy surrounding the basal expression of RIPK3, its induction, and its hepatocyte-specific contribution to APAP toxicity (31, 32). We have previously shown that RIPK3 global knockouts (RIPK3−/−) are not protected from APAP and that RIPK3 is not present in hepatocytes under basal conditions (20). We used floxed RIPK3 mice (RIPK3^flx/flx^) and targeted CRE recombinase delivery to adult hepatocytes to achieve hepatocyte-specific RIPK3 gene deletion (RIPK3^HepCKO^) using the same AAV8-TBG-CRE delivery system described above. We treated littermate RIPK3^flx/flx^ mice with AAV8-TBG-eGFP and confirmed the efficacy of viral delivery by demonstrating the presence of GFP in the control RIPK3^flx/flx^ mice (Fig. S3*A*). However, the band observed on the RIPK3 western blot in the littermate control RIPK3^flx/flx^ did not disappear with AAV8-TBG-CRE knockout, indicating that the RIPK3 detected in this band is not from a hepatocyte source and likely represents RIPK3 from liver nonparenchymal cells (NPC), such as Kupffer cells and liver sinusoidal endothelial cells (LSECs), as we have previously described using cell fractionation experiments (20). We have previously shown that RIPK3 global KOs are not protected against APAP (20). In order to definitively assess whether APAP has an effect on RIPK3 expression in hepatocytes, we repeated the *in vivo* hepatocyte targeted RIPK3 knockout and observed no difference in liver injury between RIPK3^flx/flx^ and RIPK3^HepCKO^ mice after APAP 300 mg/kg (Fig S3, *B* and *C*). Western blotting of whole-liver lysates against RIPK3 using the highly specific Genentech antibody again suggested the presence of RIPK3 originating from the NPC compartment as the band did not disappear in the AAV8 treated floxed animals (Fig. S3*D*).

### RIPK1^HepCKO^ mice have less sustained pJNK activation

The signaling cascade leading to cell death in APAP toxicity includes the activation of the MAPKs, leading to JNK phospho-activation and translocation to the mitochondria (6, 7, 8). We have previously shown that JNK was downstream of RIPK1 in experiments using antisense oligonucleotide (20). In order to determine whether JNK activation would be affected in RIPK1^HepCKO^ mice, we examined early time points post APAP.

**Figure 3:**
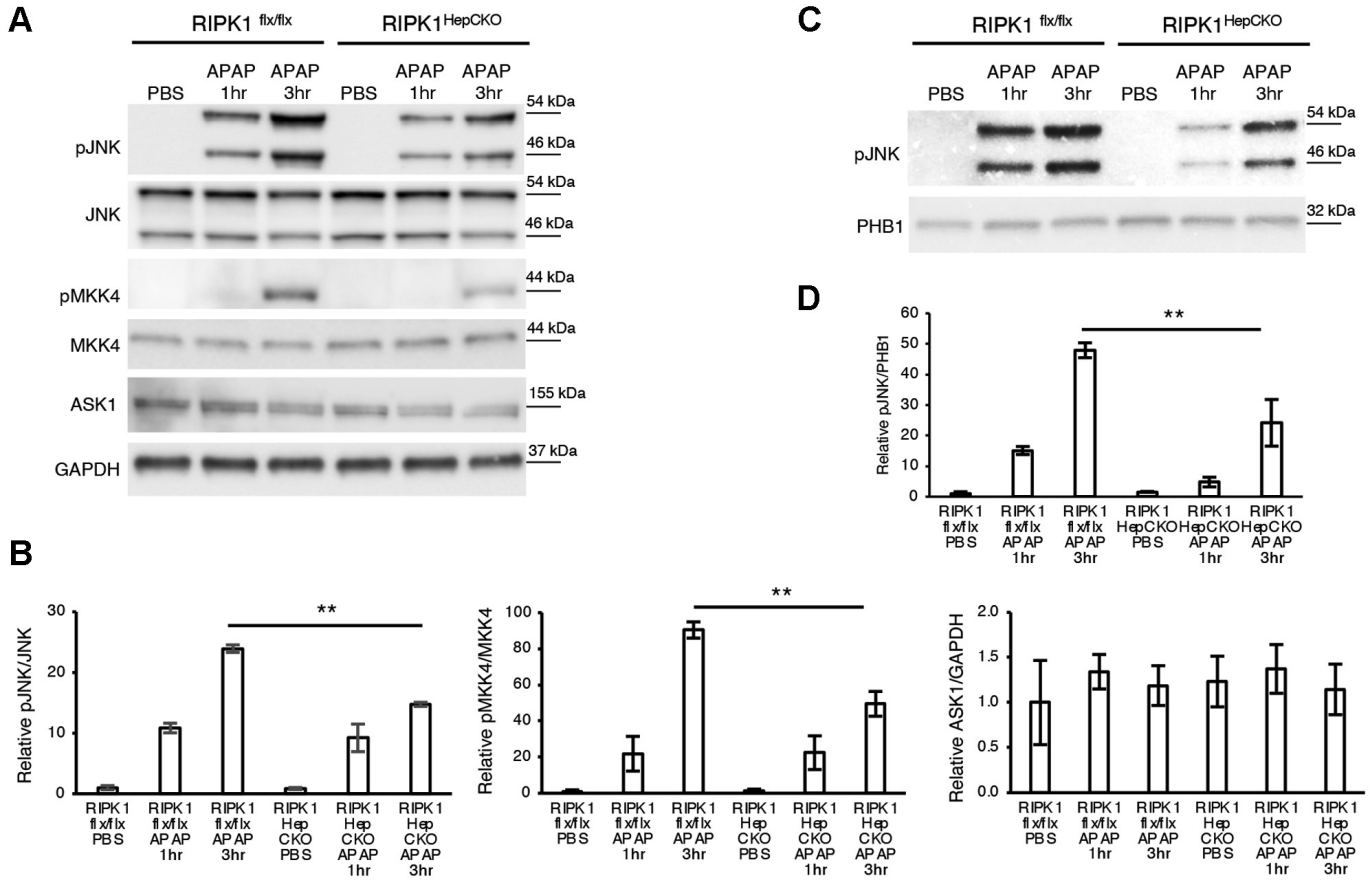
**RIPK1**^**HepCKO**^**results in dampening of JNK activation, less p-JNK translocation to the mitochondria, and decreased pMKK4**. RIPK1^flx/flx^ or RIPK1^HepCKO^ mice were fasted overnight and treated with APAP 300 mg/kg and euthanized 1 h or 3 h later. *A*, WB of liver lysates for pJNK, JNK, pMKK4, MKK4, ASK1, and GAPDH as loading control. *B*, densitometry for pJNK and pMKK4 normalized to their corresponding total protein expression and ASK1 normalized to loading control. *C*, WB of mitochondrial fraction for pJNK and loading control. *D*, densitometry of mitochondrial pJNK normalized to loading control. ∗*p* value ≤0.05 RIPK1^HepCKO^*versus* RIPK1^flx/flx^ mice and ∗∗*p* value ≤0.01 RIPK1^HepCKO^*versus* RIPK1^flx/flx^ mice. Flx, Floxed; JNK, c Jun N-terminal kinase; MKK4, mitogen-activated protein kinase 4; ASK1, apoptosis signal regulating kinase 1; RIPK1, receptor interacting protein kinase 1; GAPDH, glyceraldehyde 3-phosphate dehydrogenase; PHB1, prohibitin-1; PBS, phosphate buffered saline; APAP, acetaminophen. (N = 4/group). Results of at least three independent experiments.

Indeed, RIPK1^HepCKO^ mice displayed decreased pJNK activation post APAP (Fig. 3, *A* and *B*), as well as significantly less pJNK translocation to the mitochondria (Fig. 3, *C* and *D*).

JNK is activated through the MAPKinase cascade. The MAP2 kinase mitogen-activated protein kinase 4 (MKK4) is known to be a downstream effector of ASK1 leading to JNK activation in this pathway (9, 10). Active MKK4 (pMKK4) was also decreased in RIPK1^HepCKO^ mice compared with controls, while ASK1 protein levels remained unchanged (Fig 3, *A* and *B*). Unfortunately, we were unable to assess pASK1 protein expression, as the antibodies we tested displayed multiple nonspecific bands or were not able to detect the phosphoprotein in mouse liver.

### A20 is protective in APAP toxicity

**Figure 4:**
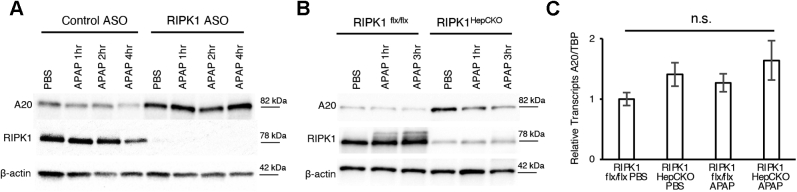
**RIPK1 knockdown and knockout increases A20 protein expression**. WT mice were treated with RIPK1 or Control ASO (50 mg/kg) five times, subsequently injected with APAP (300 mg/kg) after fasting and euthanized at 1 h, 2 h, or 4 h. *A*, representative WB of A20, RIPK1 and loading control. *B*, RIPK1^flx/flx^ or RIPK1^HepCKO^ mice were treated with PBS or APAP (300 mg/kg) after overnight fast and euthanized at 1 h and 3 h. Representative WB of A20, RIPK1 and loading control. *C*, relative A20 transcript levels normalized to TBP and RIPK1^flx/flx^ PBS (N = 5/group). ∗∗*p* value ≤0.01 RIPK1^HepCKO^*versus* RIPK1^flx/flx^. Results of at least three independent experiments. APAP, acetaminophen; ASO, antisense oligonucleotide; CTRL, control; NS, not significant; RIPK1, receptor interacting protein kinase 1; TBP, TATA box binding protein.

**Figure 5:**
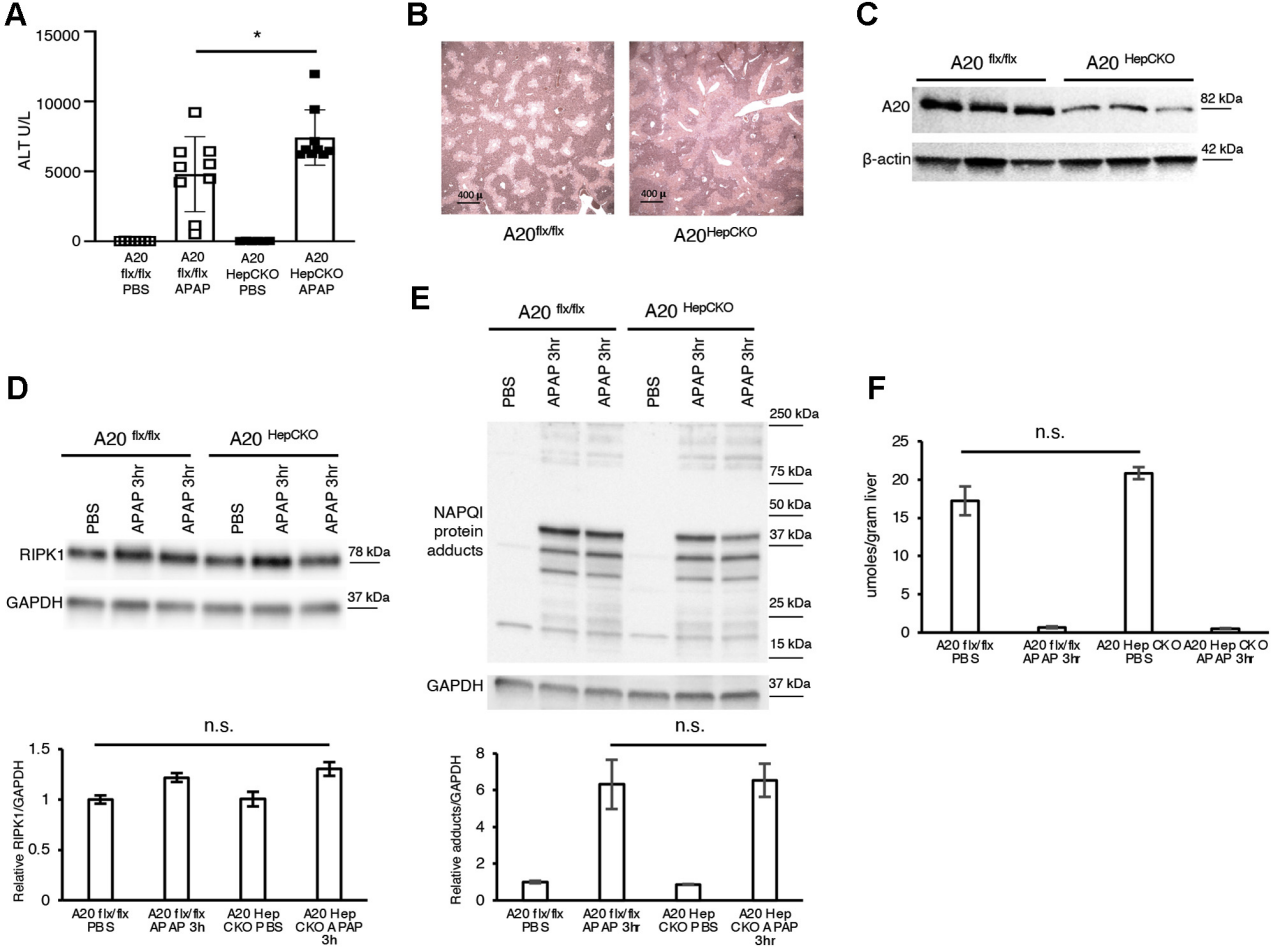
**Mice with hepatocyte-specific knockout of A20 (A20**^**HepCKO**^**) display worse liver injury following APAP compared with littermate controls (A20**^**flx/flx**^**)**. A20^flx/flx^ mice were treated with AAV8-TBG-iCRE or AAV8-TBG-eGFP for hepatocyte-specific knockout (A20^HepCKO^), after 7 days mice were fasted overnight and treated with APAP 300 mg/kg and euthanized 3 h or 24 h later. *A*, serum ALT U/L at 24 h (N = 10–11/group). *B*, representative Histology H&E (4x). *C*, representative WB of liver lysates for A20 and loading control. *D*, representative WB of RIPK1 and loading control. *E*, representative WB of NAPQI protein adducts, densitometry, and loading control (N = 4/group). *F*, GSH using a colorimetric recycling assay expressed as μmoles/gram liver. (N = 3/group). ∗*p* value ≤0.05 A20^HepCKO^*versus* A20-flx/flx mice. Results of at least three independent experiments. ALT, alanine aminotransferase; APAP, acetaminophen; Flx, floxed; GAPDH, glyceraldehyde 3-phosphate dehydrogenase; NAPQI, N-acetyl-p-benzoquinone imine; N.S., not significant; PBS, phosphate buffered saline; RIPK1, receptor interacting protein kinase-1.

A20 is a prosurvival protein that is known to interact with RIPK1 and to intersect with the MAPK pathway. A20 silencing increases JNK response, while its overexpression suppresses JNK phosphorylation and activity (26, 27, 28). ASK1 is an upstream activator of JNK in the MAPK pathway in APAP toxicity (9). A20 exerts this regulatory action on JNK by mediating ASK1 degradation (26). Since APAP-induced hepatotoxicity is associated with increased JNK phosphorylation, and RIPK1 hepatocyte KO is protective by dampening pJNK, we hypothesized that the decrease in pJNK seen in RIPK1^HepCKO^ might depend on A20. To test this hypothesis, we first examined the expression of A20 in RIPK1 antisense knockdown and RIPK1^HepCKO^ mouse livers. Indeed, A20 protein levels were increased in both (Fig. 4, *A* and *B*). However, A20 transcript levels were not significantly upregulated in RIPK1^HepCKO^ relative to RIPK1^flx/flx^ (Fig. 4*C*). In order to determine whether attenuation of A20 regulates liver damage post APAP, we generated hepatocyte-specific A20 knockout (A20^HepCKO^) mice by using adult A20 floxed mice and the same AAV8-TBG-CRE delivery system. Interestingly, A20^HepCKO^ mice demonstrated worse liver injury after APAP (Fig. 5, *A–C*), but A20 knockout had no effect on RIPK1 expression (Fig. 5*D*). A20^HepCKO^ did not affect APAP metabolism, NAPQI adduct formation, or GSH depletion compared with littermate floxed controls (Fig. 5, *E* and *F*). Notably, unlike RIPK1 knockdown or knockout, inhibiting RIPK1 kinase activity with Nec-1s did not result in increased A20 protein expression (Fig S4).

### A20 signaling in APAP necrosis is through the MAPK cascade

**Figure 6:**
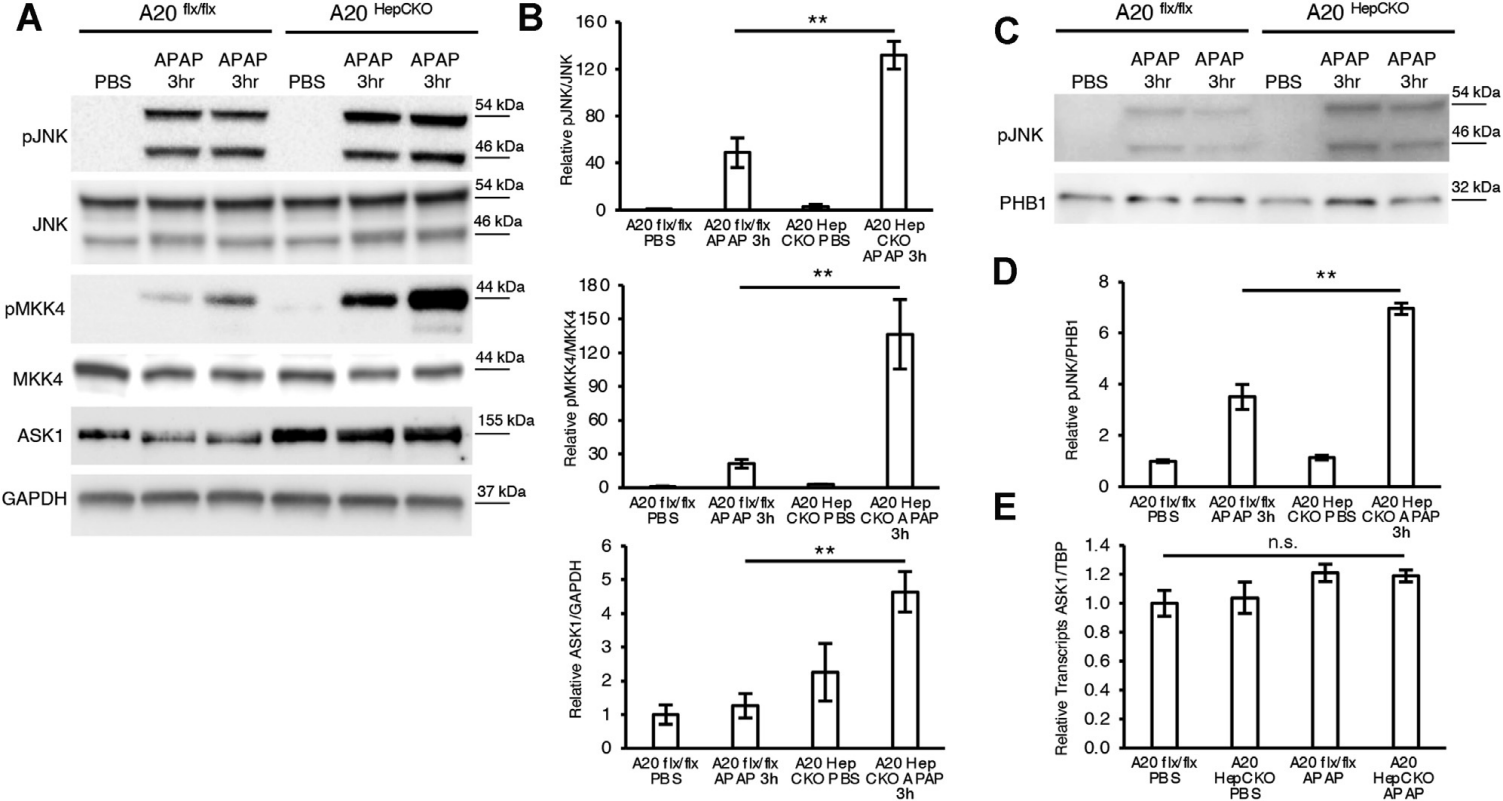
**A20**^**HepCKO**^**results in sustained JNK activation through increased ASK1 protein**. A20^flx/flx^ or A20^HepCKO^ mice were fasted overnight and treated with APAP 300 mg/kg and euthanized 3 h later. *A*, WB of liver lysates for p JNK, JNK, pMKK4, MKK, ASK1, and GAPDH as loading control. *B*, densitometry for pJNK and pMKK4 normalized to their total protein expression and ASK1 normalized to loading control. *C*, WB of mitochondrial fraction for pJNK and PHB1, loading control. *D*, densitometry is shown as mitochondrial pJNK normalized to PHB1 loading control. *E*, relative transcript levels of ASK1 normalized to TBP and RIPK1^flx/flx^ PBS (N = 5/group). ∗*p* value ≤0.05 A20^HepCKO^*versus* A20-flx/flx mice; ∗∗*p* value ≤0.01 A20^HepCKO^*versus* A20-flx/flx mice. (N = 4/group). Results of at least three independent experiments. APAP, acetaminophen; ASK1, apoptosis signal regulating kinase 1; Flx, floxed; GAPDH, glyceraldehyde 3-phosphate dehydrogenase; JNK, c Jun N-terminal kinase; MKK4, mitogen-activated protein kinase 4; PBS, phosphate buffered saline; PHB1, prohibitin-1.

Since A20 is known to degrade ASK1, thus abrogating JNK activation (26), we examined JNK expression in A20^HepCKO^ mice after APAP. Indeed, pJNK was significantly upregulated 3 h post APAP in A20^HepCKO^ compared with littermate floxed mice (Fig. 6, *A* and *B*). PJNK mitochondrial translocation was also increased (Fig. 6, *C* and *D*). Upstream of pJNK, activated MKK4 (pMKK4) was also increased in A20^HepCKO^ mice (Fig. 6, *A* and *B*). Next, we examined ASK1 and observed a significant increase in ASK1 protein levels in A20^HepCKO^ mice (Fig. 6, *A* and *B*), which was not due to an increase in mRNA levels (Fig. 6*E*). Therefore, A20 hepatocyte knockout increased total ASK1, enhancing sustained pMKK4 and pJNK activation, thus promoting cell death.

In order to ensure that hepatocyte-targeted knockdown of A20 does not sensitize the hepatocytes to a secondary wave of TNF-mediated apoptosis, we probed for cleaved caspase 3. We did not observe any cleavage of caspase 3 post APAP in A20^HepCKO^ mouse livers nor in RIPK1^HepCKO^ livers (Fig. S5, *A* and *B*).

### A20 coimmunoprecipitates with RIPK1 and ASK1

**Figure 7:**
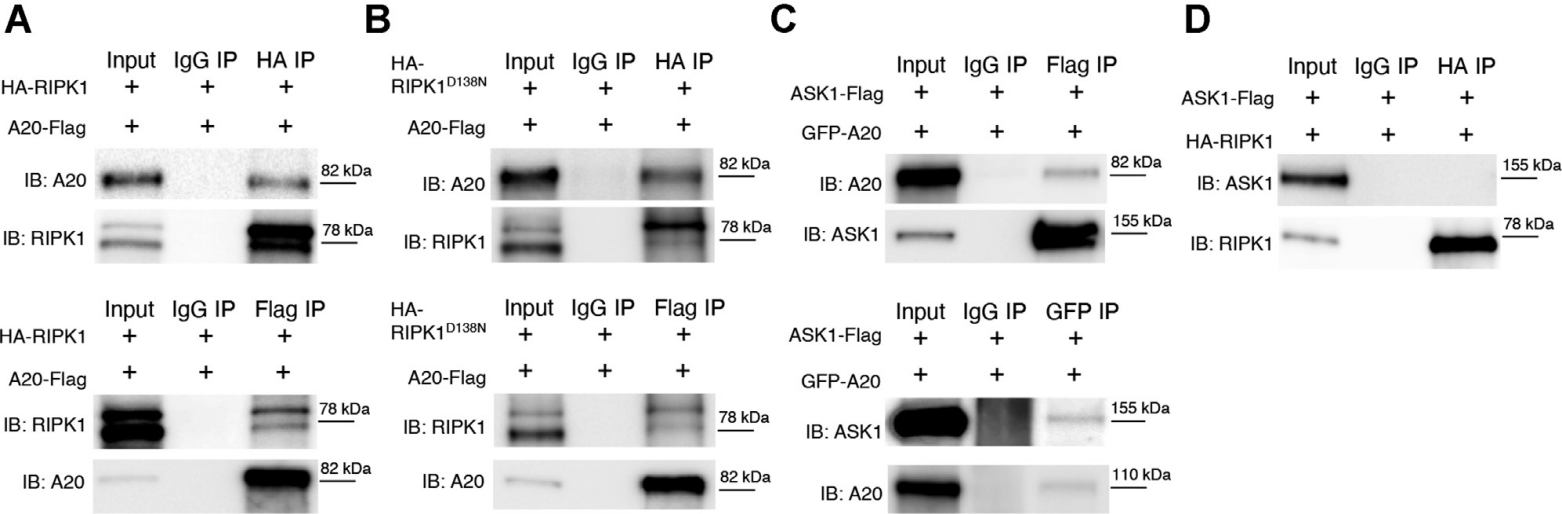
**A20 Coimmunoprecipitates with RIPK1 and ASK1 *in vitro***. *A*, HEK293T cells were cotransfected to overexpress HA-RIPK1 and A20-Flag for 24 h. Cells were harvested and IP was performed with HA Tag, Flag Tag, or isotype matched control IgG. Membranes were immunoblotted for A20 and RIPK1. *B*, HEK293T cells were cotransfected to overexpress the kinase dead mutant HA- RIPK1^D138N^ and A20-Flag for 24 h, and subsequently lysates were IPed with HA Tag, Flag Tag, or isotype control IgG. Membranes were immunoblotted for A20 and RIPK1. *C*, HEK293T cells were cotransfected to overexpress GFP-A20 and ASK1-Flag for 24 h, and subsequently IP was performed with GFP Tag, Flag Tag, or isotype control IgG. Membranes were immunoblotted for A20 and ASK1. *D*, HEK293T cells were cotransfected to overexpress ASK1-Flag and HA-RIPK1 or control IgG for 24 h. Lysates were IPed with HA tag and immunoblotted against ASK1and RIPK1. Results of at least three independent experiments.

RIPK1 and A20 are known to associate in Complex I after TNF stimulation. However, little is known about their association outside of TNF signaling in the context of APAP toxicity and necrosis. Since RIPK1 hepatocyte-specific KO increased A20 levels, and this was not transcriptional, we explored whether there was an interaction at the protein level. We generated a cell culture model system where we overexpressed the tagged proteins HA-RIPK1 and A20-Flag in HEK293T cells. After 24 h, we immunoprecipitated (IP) either Flag or HA tag and probed for both proteins to test whether A20 and RIPK1 co-IP. Indeed, pull-down of Flag-tagged A20 resulted in co-IP of HA-RIPK1. The reverse was also true, HA-tagged RIPK1 IP resulted in its pull-down of Flag-A20 as well (Fig. 7*A*). Therefore, RIPK1 and A20 directly interact.

As shown previously (Fig. 1), the kinase function of RIPK1 is dispensable during APAP toxicity. Therefore, we examined if the kinase function of RIPK1 was necessary for its interaction with A20. We thus generated an HA-tagged RIPK1-D138N construct by creating a GAC (aspartic acid) to AAC (asparagine) point mutation at position 138 and proceeded to repeat our previous co-IP. We observed no difference between the ability of WT RIPK1 and kinase dead RIPK1^D138N^ in pulling down A20 (Fig. 7*B*). The reverse was also true as Flag-A20 was able to pull down kinase dead HA-RIPK1^D138N^ (Fig. 7*B*). These results were further validated in the hepatocyte-derived cell line, Huh7 (Fig. S6, *A* and *B*). We confirmed the RIPK1-A20 interaction seen in our model was not due to binding artifact between the tags by transfecting the prey without the bait and pulling down the two tags, HA and Flag, separately. (Fig. S6*C*). Although RIPK1 levels do not change in the A20^HepCKO^ mice, it is possible that A20, an E3 ligase and deubiquitinase, affects RIPK1 posttranscriptionally. We therefore IPed RIPK1 in the presence and absence of A20 and indeed noticed less polyubiquitin stranding with A20 overexpression (Fig S7). However, more detailed ubiquitination studies and confirmation of these interactions *in vivo* are needed to assess the type of ubiquitin modification.

Lastly, since A20^HepCKO^ enhanced expression of ASK1 *in vivo*, and this was not through increased ASK1 transcription, we wanted to explore the posttranscriptional effects of A20 on ASK1. A20 has been shown to bind to and regulate ASK1 by either degradation or inhibition (26, 28). Thus, we overexpressed GFP-A20 together with ASK1-Flag in HEK293T cells. Indeed, ASK1 Co-IPed with A20 and vice versa, A20 pulled down ASK1 (Fig. 7*C*). However, we did not find any direct interaction between RIPK1 and ASK1 (Fig. 7*D*).

### RIPK1 prevents the A20-ASK1 interaction

**Figure 8:**
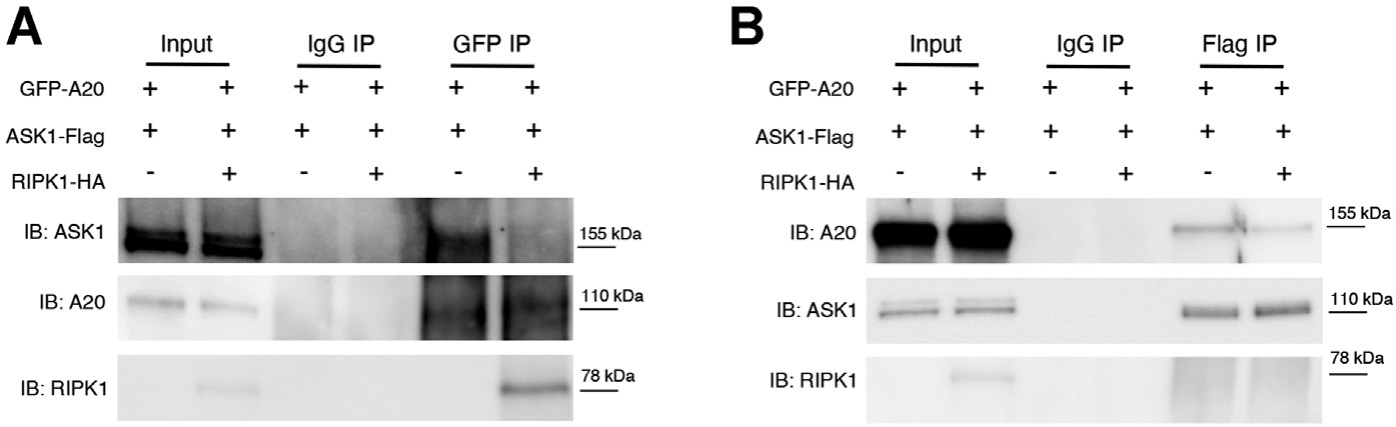
**RIPK1 interferes with the A20-ASK1 interaction by binding to and sequestering A20 *in vitro***. *A*, HEK293T cells were cotransfected to overexpress GFP-A20 and ASK1-Flag either with HA-RIPK1 or control vector for 24 h. Cells were harvested and IP was performed with GFP Tag or isotype-matched control IgG. Membranes were immunoblotted for ASK1, A20, and RIPK1. *B*, HEK293T cells were cotransfected to overexpress GFP-A20 and ASK1-Flag either with HA-RIPK1 or control vector for 24 h, and subsequently lysates were IPed with Flag Tag or isotype-matched control IgG. Membranes were immunoblotted for A20, ASK1, and RIPK1.

So far, we have observed that A20 binds both RIPK1 and ASK1, and when RIPK1 is absent, A20 is upregulated through a posttranscriptional mechanism. Therefore, we hypothesized that binding of A20 to RIPK1 sequesters A20 to minimize the A20-mediated degradation and/or inhibition of ASK1, and when RIPK1 is absent, A20 is free to bind ASK1. To test this, we cotransfected HEK293T cells to overexpress GFP-A20 and ASK1-Flag in the presence or absence of HA-RIPK1. We show that in the presence of RIPK1, GFP-tagged A20 pulls down less ASK1 than in the absence of RIPK1 (Fig. 8*A*), and Flag-tagged ASK1 pulls down less A20 when RIPK1 is present than when it is absent (Fig. 8*B*). As seen previously, A20 is able to pull down RIPK1, while ASK1 is not. Therefore, when RIPK1 is depleted, increased A20 is free to associate with ASK1, thus affecting downstream signaling such as decreasing pMKK4 and dampening JNK activity, resulting in protection against APAP necrosis.

## Discussion

Drug-induced liver injury is a significant cause of liver disease. APAP overdose, which results in massive hepatocyte necrosis, is the number one cause of acute liver failure in the United States (1). APAP necrosis is the result of ROS generation from APAP's toxic metabolite NAPQI, which depletes GSH and results in mitochondrial membrane collapse (2). Multiple signaling events lead to this form of necrotic cell death, which is thus considered to be a form of regulated, and not accidental, necrosis (33). The ROS accumulation resulting from mitochondrial toxicity activates the MAPKs, including ASK1, which leads to phospho-activation of downstream kinases such as MKK4 and ultimately, JNK (11). APAP-induced RCD occurs through sustained pJNK activation, as interference with pJNK signaling at various stages has been shown to be protective. RIPK1 is a key signaling protein with kinase-dependent and -independent functions, which is known to mediate apoptosis, necrosis, necroptosis, inflammation, and survival (12). Its kinase function mediates its pronecroptotic and proapoptotic properties, while its platform function mediates MAPK and NFκB activation (12). Necroptosis involves RIPK1 and RIPK3 oligomerization leading to the phospho-activation of MLKL by RIPK3. While this form of cell death has garnered much attention in recent years, its occurrence in hepatocytes, which lack RIPK3 expression, has been controversial, especially in the context of APAP necrosis (32). We have previously shown that knockdown of RIPK1 using ASO is protective against APAP RCD upstream of JNK (20). This has been confirmed by others using siRNA knockdown of RIPK1 (21). Additionally, we previously showed that while RIPK1 knockdown was protective, RIPK3 or MLKL knockout was not (20). We therefore concluded that RIPK1 participates in the hepatocyte cell death pathway in the APAP model independent of its role in the necrosome. Here, we set out to elucidate whether the kinase-dependent or -independent function of the RIPK1 protein mediates APAP toxicity. We treated RIPK1^D138N^ kinase dead knock-in mice with APAP and found no difference in injury compared with WT controls. However, ASO-mediated knockdown of the kinase dead protein in these mice was protective, indicating that the platform function of RIPK1 participates in APAP toxicity.

Since ASO treatment knocks down proteins in the entire liver cell type population, we wanted to determine whether our previous finding showing protection against APAP with RIPK1 ASO was due to the hepatocyte knockdown of the protein, as well as to exclude any off-target effects of the ASO. Using AAV8 viral delivery of CRE recombinase, we generated conditional knockouts lacking hepatocyte RIPK1, *i.e.*, RIPK1^HepCKO^. Indeed, RIPK1^HepCKO^ mice were also protected against APAP. Mechanistically, we observed decreased pJNK activation in liver lysates of RIPK1^HepCKO^ mice after APAP, as well as significantly less pJNK translocation to the mitochondria.

TNFAIP3 or A20 is a prosurvival protein that has been shown to interact with ASK1 and promote ASK1 degradation, thereby affecting JNK activation (26). Since hepatocyte-specific RIPK1 knockout abrogates pJNK as well, and RIPK1 and A20 are known to interact, we investigated whether RIPK1 deficiency affects A20 expression following APAP RCD. Both RIPK1 knockdown and knockout resulted in increased expression of A20 at the protein level, but not at the mRNA level. The mechanism for stabilization of A20 in the absence of RIPK1 is not known; however, proteasomal degradation of A20 has been described in other contexts (34, 35). How RIPK1's absence inhibits proteasomal degradation and turnover of A20 remains to be explored and is beyond the scope of the current work. Next, we knocked out A20 in adult hepatocytes using the same viral CRE delivery system and confirmed that the mice had no baseline liver inflammation. Interestingly, after treatment with APAP, the A20^HepCKO^ mice demonstrated more liver injury compared with floxed littermate controls. To exclude the possibility that A20 knockout sensitizes the hepatocytes to a secondary wave of inflammation-induced apoptosis following APAP, we examined lysates for caspase-3 and found no evidence of caspase-3 cleavage, thus excluding the possibility of a switch to apoptosis. A20 ^HepCKO^ had no effect on RIPK1 expression.

A20 has been shown to bind to ASK1 and promote its degradation (26, 27, 28). We therefore probed MAPK proteins and observed that A20 knockout significantly increased basal ASK1 protein, pMKK4, as well as pJNK. Therefore, we hypothesize that in the APAP model of RCD, A20 regulates ASK1 expression, thus ultimately affecting pJNK activation and mitochondrial translocation. This finding was consistent with published work showing that overexpression of A20 inhibited ASK1 activation through binding and K63 deubiquitination without promoting degradation (28). Although knockout of A20 upregulated ASK1 protein, it did not affect transcript levels. Unfortunately, we were unable to obtain a working mouse pASK1 antibody that was sensitive and specific enough to see if ASK1 phosphorylation is inhibited by RIPK1 knockout. However, since MKK4 activation was decreased in RIPK1^HepCKO^, we believe this is consistent with A20-regulated inhibition upstream of MKK4 and JNK. Further work will be required to assess this.

Since A20 levels increased with RIPK1 knockout in hepatocytes and this was not transcriptional, we wanted to determine if there was an interaction between RIPK1 and A20 at the protein level. These proteins have been shown to interact under certain conditions such as TNF or LPS stimulation (3, 36). We conducted a series of Co-IP experiments and observed that, indeed, A20 does interact with RIPK1. The interaction of A20 and RIPK1 was not dependent on RIPK1 kinase function, as a mutant kinase dead RIPK1 protein RIPK1^D138N^ was also able to pull down A20 and vice versa. We next conducted experiments where we expressed both A20 and ASK1 and were able to Co-IP both proteins. However, we were unable to detect any direct interaction between RIPK1 and ASK1.

**Figure 9:**
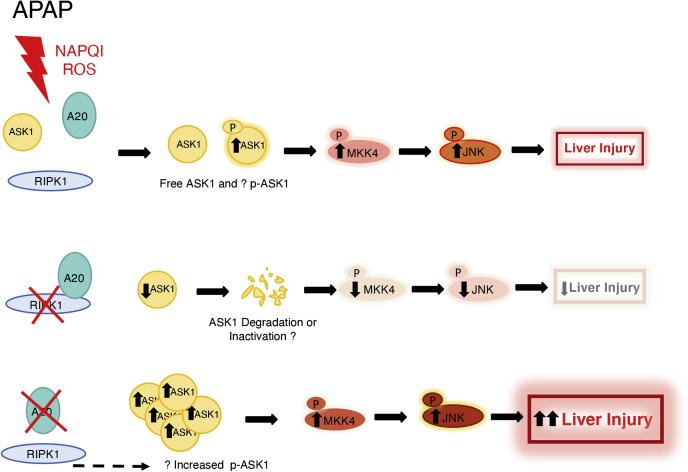
**Proposed signaling mechanism. After APAP, ASK1 is activated (pASK1) and results in the activation of MKK4 (pMKK4), pJNK, and liver injury**. Knockout of RIPK1 in hepatocytes leads to increased A20 levels, which allow for A20 to bind, sequester, and possibly inactivate or degrade ASK1, thus yielding a downregulation of pMKK4 and pJNK, which ultimately result in an attenuation in liver injury. Conversely, in hepatocyte-specific A20 knockout livers, ASK1 protein is increased, leading to phospho-activation of MKK4 and sustained pJNK activation, which result in increased liver injury.

We tested this hypothesis by designing cotransfections of HEK293T cells with GFP-A20 and ASK1-Flag in the presence of HA-RIPK1 or an empty vector. We found that in the presence of RIPK1, both GFP-A20 and ASK1-Flag pull each other down less efficiently than when RIPK1 is absent. Since overexpression of RIPK1 prevented this interaction and *in vivo* knockout of RIPK1 increased A20 protein levels, we therefore suggest a model in which the absence or depletion of RIPK1 allows for more association of A20 to ASK1, potentially causing its inactivation and/or degradation and leading to an attenuation of pJNK activation and decreased liver injury. In the absence of A20, there is an increase in ASK1 protein, leading to increased pMKK4 and pJNK and more liver injury (Fig. 9). However, further work is required to explore the A20-mediated regulation of ASK1 and RIPK1-mediated regulation of A20 in this mode of cell death.

Our study is unique in its use of multiple transgenic models and importantly, in using an adult hepatocyte-specific knockout model. Furthermore, it is the first reported association of RIPK1 and A20 in APAP RCD and acute liver injury. Our results regarding the protective role of RIPK1 knockdown or knockout upstream of JNK confirm previous studies (20, 21). However, this is the first report of the adult hepatocyte-specific RIPK1 knockout in the APAP model. Our results differ from one previous report that showed embryonic liver parenchymal cell-specific knockout of RIPK1 did not protect mice from APAP (37). Currently, the explanation for these conflicting results is not clear. Given the vital role of RIPK1 in development, one potential confounding factor in the embryonic liver parenchymal cell RIPK1 knockouts is unforeseen compensatory developmental effects that might affect the outcome. This is a phenomenon well described in some strains of embryonic knockout mice (38). While the embryonic liver knockout of RIPK1 is not lethal, global RIPK1 knockout mice die soon after birth (39). We therefore decided to use an inducible adult RIPK1 knockout model and conducted experiments within 10 days of knockout, thereby minimizing the possibility of compensatory effects, although not eliminating them completely. We acknowledge that adult inducible transgenic models are not perfect, but believe they are currently the best tools available to study the impact of a single gene. Additionally, we note that A20 deletion may affect sensitivity to TNF-induced liver apoptotic cell death (25). Although TNF is believed to not have a role in APAP toxicity in WT mice, this might contribute to exacerbation of APAP toxicity in A20^HepCKO^ despite our finding of no caspase 3 activation in this context.

The posttranslational modifications of RIPK1, particularly its ubiquitination status, dictate its function (40). Downstream of the TNF receptor, A20, which is both an E3 ligase and a deubiquitinase, is known to both deubiquitinate RIPK1 (K63) and ubiquitin ligate the protein (K48), targeting it for degradation (41). Although, we did not see an effect on total RIPK1 protein (no degradation in the A20^HepCKO^), it is possible that nondegradative ubiquitin modifications could contribute to our findings. However, given the complexity surrounding RIPK1's posttranslational modifications, and the various possible ubiquitin linkages (40), we were unable to study these within the scope of the current work. Further detailed *in vivo* and *in vitro* ubiquitination experiments in the presence and absence of A20 with mutagenesis of lysine residues *in vitro*, as well as *in vivo* confirmatory studies in A20^HepCKO^ mice, are warranted to explore these modifications in the absence of hepatocyte A20.

In summary, RIPK1 knockout attenuated MAPK signaling through upregulation of A20 and protects against APAP toxicity. We hypothesize that A20 exerts this effect through its interaction with ASK1, which likely results in the degradation or inhibition of the protein and dampening of the sustained activation of downstream signaling proteins such as MKK4 and JNK. Given the importance of MAPK signaling in the liver, it will be interesting to assess whether the RIPK1-A20 interaction contributes to other models of liver injury.

## Experimental procedures

### Reagents

For western blot (WB) analyses, we used antisera to RIPK1, JNK, P-JNK, Glyceraldehyde-3-phosphate dehydrogenase (GAPDH), β-actin, A20, ASK1, GFP, Flag Rabbit, DYKDDDK Tag, and Prohibitin-1 (PHB1) from Cell Signaling and anti-Flag M2 mouse monoclonal antibody from Sigma. Monoclonal RIPK3 antibody was provided by Dr Kim Newton (Genentech). For Coimmunoprecipitation (Co-IP) we used, anti-Flag-M2 affinity agarose gel (Sigma), anti-HA tag mouse monoclonal antibody (Abcam), and anti-GFP rabbit monoclonal antibody (Cell Signaling Technologies) and normal rabbit and mouse IgG (Santa Cruz Biotechnology). Dimethyl Sulfoxide (DMSO) and APAP were obtained from Sigma. The AAV8-TBG-iCRE and AAV8-TBG-eGFP viral vectors were from Vector Biolabs. N-acetyl-p-benzoquinone imine (NAPQI) adduct antisera was provided by Dr Laura James at the University of Arkansas. RT-qPCR reagents and probes were from Applied Biosystems. SuperScript IV Vilo Master Mix (Invitrogen) was used to achieve cDNA synthesis. Necrostatin-1 (Nec-1) was from Calbiochem and Nec-1s from BioVision. Liver tissues for RNA extraction were snap-frozen in liquid nitrogen and stored at –80 °C until RNA was extracted using the Trizol (Invitrogen) method as previously described (29). Serum levels of alanine aminotransferase (ALT) were assessed using a kit from Teco Diagnostics. For cell necrosis quantification, Hoechst 33,258 was purchased from Invitrogen and Sytox Green from Thermo Fisher.

### Animals

Ten to 12-week-old male mice weere used. WT C57BL/6n mice were from Envigo. RIPK1^D138N^, RIPK1^flx/flx^, RIPK3^flx/flx^ mice (all C57BL/6n) were provided by Dr Kim Newton from Genentech. A20^flx/flx^ mice (on a C57BL/6j background strain) were provided by Dr Ling Shao. All animals were housed in an environmentally controlled room with 12 h light/dark cycle and allowed free access to food and water. The experimental protocol was approved by the Institutional Animal Care and Use Committee at USC. All animals received humane care according to the criteria outlined in the Guide for the Care and Use of Laboratory Animals prepared by the National Academy of Sciences. For conditional RIPK1, RIPK3, and A20 knockout, RIPK1 ^flx/flx^, RIPK3 ^flx/flx^ and A20 ^flx/flx^ mice were given one single tail vein injection of 2 × 10^11^ g.u. of AAV8-TBG-iCRE, Vector Biolabs. (RIPK1^HepCKO^, RIPK3^HepCKO^ and A20^HepCKO^ groups). AAV8-TBG-eGFP was given to littermate controls at the same dose (RIPK1-flx/flx, RIPK3-flx/flx and A20-flx/flx groups). All results include data from at least four independent experiments.

APAP was dissolved in warm phosphate buffered saline (PBS) at 55 °C and cooled to 37 °C before intraperitoneal (IP) injection of overnight-fasted mice at a dose of 300 mg/kg for transgenic mice or 500 mg/kg in experiments with DMSO, as previously described (20). In *in vivo* experiments, Nec-1s was dissolved in 10 % DMSO and PBS (1 mg in 200 μl of DMSO diluted with 1800 μl of PBS). Nec-1s (4 mg/kg) was injected intraperitoneally 45 min prior to APAP injection (500 mg/kg). The same volume of DMSO/PBS was injected into control animals. Serum alanine aminotransferase (ALT) was measured according to manufacturer's protocol.

### Antisense treatment

Antisense (ASO) targeting mouse RIPK1, CTCCATGTACTCCATCACCA, and control scrambled oligonucleotide, CCTTCCCTGAAGGTTCCTCC, were provided by Ionis Pharmaceuticals. Oligonucleotides were synthesized as 20-nt uniform chimeras containing five nuclease-resistant *2′-O-* methoxyethylribose-modified phosphorothioate residues on the 5′ and 3′-ends, flanking a 2′-deoxyribonucleotide/phosphorothioate region, which supports RNase H1-based cleavage of the targeted mRNA. WT and RIPK1^D138N^ male mice were treated with ASO every other day at the dose of 50 mg/kg for 10 days prior to APAP injection (300 mg/kg for WT and 500 mg/kg for WT and RIPK1^D138N^, which were pretreated with Nec1s or DMSO vehicle).

### Hepatocyte isolation and culture

Freshly isolated hepatocytes from RIPK1^D138N^ mice were separated by percoll (Sigma) centrifugation to remove debris and remaining nonparenchymal cells as previously described (20). Three hours after plating of the hepatocytes, APAP 30 mM dissolved in fresh prewarmed DMEM/F12 culture medium was added. After 2 h exposure to APAP, the culture media was changed and the APAP was removed; then hepatocytes were treated for 24 h with either Nec-1 (50 μM), Nec-1s (50 μM) or DMSO as control. After 24 h of treatment with inhibitors, cells were double-stained with Hoechst 33,258 (Invitrogen) and Sytox Green (Thermo). Quantification of total and necrotic cells (Sytox Green positive) was performed by counting a minimum of 1000 cells in ten different fields using image J, as previously described (15). Cell viability was assessed at 24 h after exposure.

### Isolation of liver mitochondria and culture

Mitochondria were isolated by differential centrifugation as previously described (15). Briefly, the livers were homogenized in H-medium (250 mM sucrose, 20 mM HEPES, 1 mM EDTA, 1 mM EGTA, plus protease, and phosphatase inhibitors). The homogenate was centrifuged at 800*g* for 10 min twice, the resulting supernatant from the second spin was centrifuged at 8,000*g* for 15 min, at which point the supernatant (cytoplasm) was removed and the pellet (crude mitochondria) was washed with H-medium and the centrifugation was repeated. The mitochondria in the final pellet were resuspended in RIPA buffer (containing 50 mM Tris-HCl (pH 8), 150 mM NaCl, 0.5% deoxycholate, 1% NP-40, 0.1% sodium dodecyl sulfate, 1 mM EGTA, and 1 mM EDTA) supplemented with phosphatase and protease inhibitor cocktails from Sigma for WB analysis.

### Western blot

Liver lysates were prepared by homogenizing 80 mg liver tissue in 1 ml RIPA buffer or H-Medium (see above) with phosphatase and protease inhibitor cocktails from Sigma with a dounce homogenizer. The samples were then centrifuged at 14,000*g* for 5 min to pellet DNA and the supernatants were collected. The protein concentrations were measured and 30 μg of protein was treated with β-mercaptoethanol/Laemmeli loading buffer prior to gel electrophoresis. Subsequently, proteins were transferred to polyvinylidene difluoride membranes and blots were blocked with 5% (w/v) nonfat milk or 5% bovine serum albumin dissolved in Tris-buffered saline with Tween 20. The blots were then incubated with the desired primary and secondary antibodies. Finally, the proteins were detected by Luminol ECL reagent (Thermo) using a Bio-Rad ChemiDoc MP Imaging System (Bio-Rad). Densitometry was quantified using Image Lab Software (Bio-Rad). All WBs and densitometry shown are of representative samples from at least four independent experiments.

### Real-time quantitative polimerase chain reaction (RT-qPCR)

For RT-qPCR quantification, livers were excised, rinsed thoroughly of blood, snap-frozen in liquid nitrogen, and stored at –80 °C until RNA extraction. RNA was extracted using the Trizol method and 2 μg of RNA was reverse-transcribed using the Supercript IV Vilo Master Mix according to manufacturer's protocol in a final reaction volume of 20 μl. The real-time qPCR was performed using TaqMan probes against TBP (Mm01277042_m1), ASK1 (Mm00434883_m1), and A20 (Mm00437121_m1) according to manufacturer's protocol in a final volume of 20 μl using TaqMan Fast Advanced Master Mix and a Step One Plus PCR system (Applied Biosystems). For all assays, there were at least five samples/groups assayed in duplicate. Threshold cycle (Ct value) was determined using the Step One Plus software, and the Ct value of the gene of interest was normalized to the Ct value of its own internal control gene (TBP). ΔΔCt values normalized to the control group are reported. PCR controls consisted of the reaction cocktail without reverse transcriptase and H_2_O instead of cDNA tested by RT-qPCR.

### GSH measurement

Total GSH levels were assessed in total liver homogenates using a Glutathione Assay Kit (Cayman Chemical) according to manufacturer's protocol.

### Histological analysis

Livers were removed, fixed with 10% buffered formalin, embedded in paraffin, and cut into 5 μm-thick sections. All specimens were stained with hematoxylin-eosin (H&E) and evaluated under light microscopy.

### Subcloning

Human RIPK1 was purchased from Addgene (clone # 78,842) and was subsequently tagged with three C-terminal HA tags and subcloned into pCDNA 3.3 using a TOPO TA cloning kit (Thermo Fisher Scientific) according to manufacturer's protocols to yield our 3HA-pCDNA3.3-RIPK1 construct. The hRIPK1-D138N clone was achieved by using QuickChange II Site-Directed Mutagenesis Kit (Agilent Technologies) to create a GAC (aspartic acid) to AAC (asparagine) point mutation at position 138 according to manufacturer's protocol using our previously cloned 3HA-pCDNA3.3-hRIPK1 construct as the template. Our pCMV-hA20-Flag construct was courtesy of Dr Ling Shao, Keck School of Medicine of the University of Southern California, while the pEGFP1-C1-hA20 and pCMV-hAsk1-Flag constructs were purchased from Addgene (clone # 22141 and 47106, respectively). Once the sequences were verified, the clones were amplified using an EndoFree Plasmid Maxi Kit (Qiagen).

### Coimmunoprecipitation (CO-IP)

For Co-IP, either Huh7 or HEK-293T (HEK) cells were overexpressed with our constructs and harvested either at 24 h (for HEK cells) or at 48 h (for Huh7 cells). Cells were harvested in an IP buffer containing: 20 mM Tris, pH 7.5, 150 mM NaCl, 5 mM EDTA, 5 mM beta-glycerolphosphate, 5 mM NaF, 10% Glycerol and 0.1% BME, 0.5% Triton, and protease and phosphatase inhibitors (1:100) by scraping the cells and then incubating for 1 h at 4 °C with continuous rotation. Precleared protein (with protein A/G plus agarose beads) was then incubated overnight at 4 °C with continuous rotation with anti-Flag-M2 affinity agarose gel, anti-HA tag mouse monoclonal, or anti-GFP rabbit monoclonal antibodies. Normal rabbit IgG and normal mouse IgG served as isotype-matched controls. The next day protein A/G agarose beads (Santa Cruz Biotechnology) were used to pull down overexpressed proteins. The Co-IP products were then subjected to gel electrophoresis and probed for pull-down of self as well as of molecular partners *via* WB.

### Statistical analysis

Values were described as mean ± standard error of the mean (SEM). Student's *t*-test was used to compare differences as appropriate. *p*-value < 0.05 was defined as statistically significant. One-way analysis of variance test was used to compare group mean differences as appropriate. Post-hoc analysis for multiple comparisons within groups was performed with the Holm–Sidak test. Statistical analyses were performed using R 3.5.2 (R Foundation for Statistical Computing).

## Data availability

All data are located within the article.

## Conflicts of interest

Dr Mariam Aghajan is an employee of IONIS pharmaceuticals. All other authors declare that they have no conflicts of interest with the contents of this article.
