# Supplementary material for: Interaction of RIPK1 and A20 modulates MAPK signaling in murine acetaminophen toxicity
Source: J Biol Chem. 2021 Jan 16;296:100300. doi: 10.1016/j.jbc.2021.100300 (PMC7948960; doi:10.1016/j.jbc.2021.100300)
Supplement: Figures S1 to S7 [file mmc1.pdf]

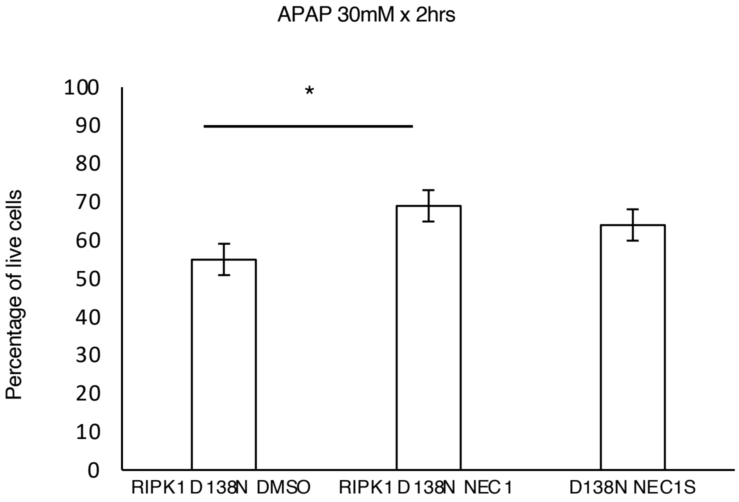

Fig. S1. Treatment with Nec1, but not Nec1s, protects primary mouse hepatocytes (PMHs) isolated from RIPK1^{D138N} mice against APAP toxicity *in vitro*. PMHs were isolated from RIPK1^{D138N} and plated on collagen coated plates for 2 hrs. The media was then changed and 30 mM APAP was added for 2 hr. Subsequently, APAP was removed and 50 μM inhibitors were added in fresh media. Images were taken 24 hrs later. % viable cells as averaged per 10 high power fields/condition. N=3 individual experiments. RIPK1-Receptor Interacting Protein Kinase 1; GAPDH-Glyceraldehyde 3-phosphate dehydrogenase; RIPK1^{D138N}- RIPK1 kinase dead knock-in mice, APAP-acetaminophen; DMSO- dimethyl sulfoxide; NEC1- necrostatin-1; NEC1S- necrostatin-1s.

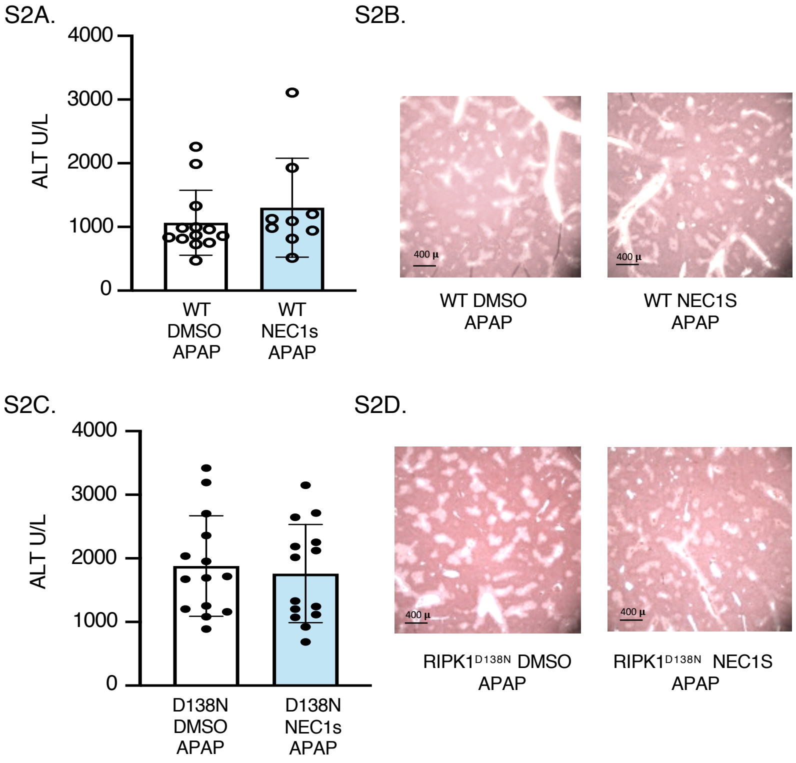

Fig. S2. Nec1s does not protect RIPK1^{D138N} mice against APAP toxicity *in vivo*. For A and B, WT mice were fasted overnight and treated with vehicle (DMSO) or Nec1s 45 min prior to APAP 500mg/kg and euthanized 24 hrs later. (A) Serum ALT (U/L). (B) Representative histology H&E (4x). For C and D, RIPK1^{D138N} were fasted overnight and treated with vehicle (DMSO) or Nec1s 45 min prior to APAP (500mg/kg) and euthanized at 24 hrs. (C) Serum ALT (U/L). (D) Representative histology H&E (4x). N=9-13/group. WT- wild-type; DMSO-dimethyl sulfoxide; APAP- acetaminophen; NEC1S- necrostatin-1s.

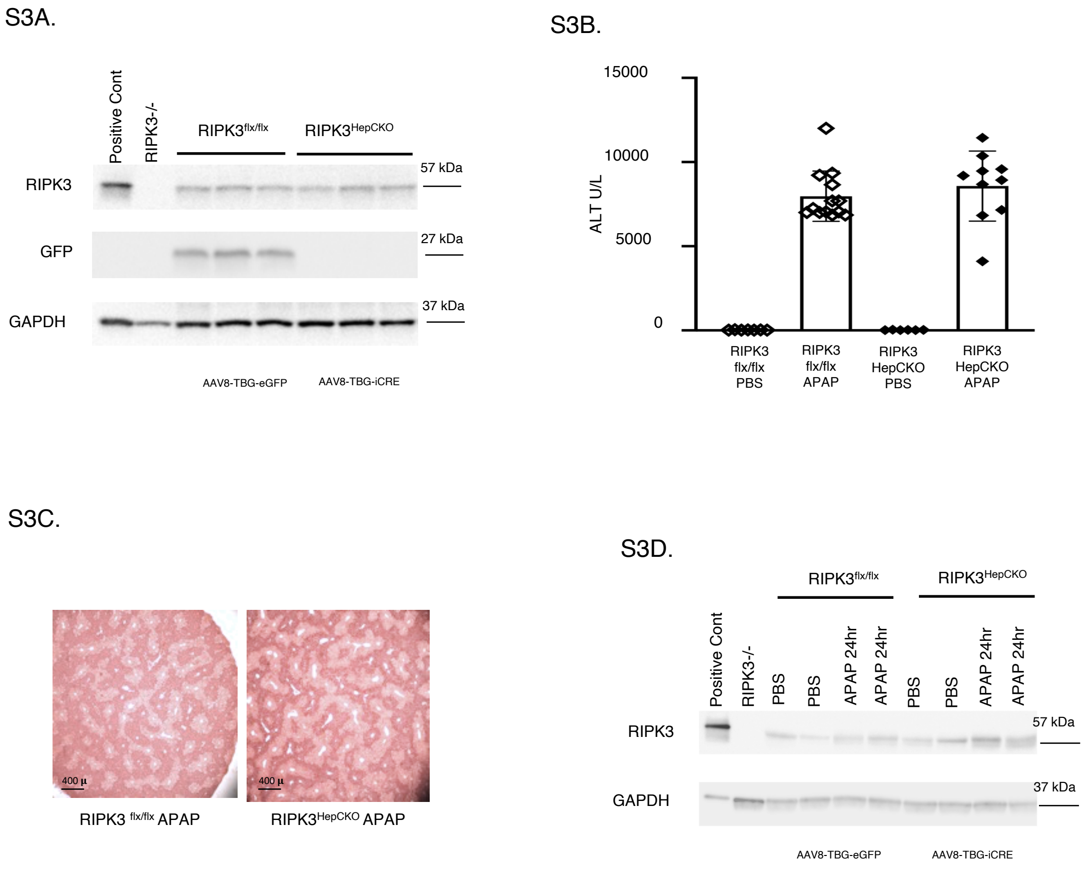

Fig. S3. Hepatocyte-specific RIPK3 knockout (RIPK3^{HepCKO}) does not protect against APAP. (A) RIPK3$^{flx/flx}$ mice were treated with AAV8-TBG-iCRE or AAV8-TBG-eGFP for hepatocyte specific targeting of CRE. WB of GFP was performed to confirm viral delivery of GFP. The RIPK3 band observed is from the liver non-parenchymal cell (NPC) population. Positive control is WT spleen and negative control is global RIPK3-/- liver. (B) RIPK3$^{flx/flx}$ mice were treated with AAV8-TBG-iCRE or AAV8-TBG-eGFP for hepatocyte specific targeting of CRE. Ten days later mice were fasted overnight and treated with PBS or APAP 300mg/kg and euthanized 24 hrs later. Serum ALT (U/L) (N=10/group) (C) Representative Histology H&E (4x). (D) WB with Genentech RIPK3 monoclonal Ab and loading control without and with APAP (300mg/kg) for 24hrs. Positive control is WT spleen and negative control is global RIPK3-/- liver. Results of at least three independent experiments. ALT- alanine aminotransferase; PBS- phosphate buffered saline; APAP-acetaminophen; Flx-Floxed; GFP-Green Fluorescent Protein; GAPDH-Glyceraldehyde 3-phosphate dehydrogenase; RIPK3-Receptor Interacting Protein Kinase 3.

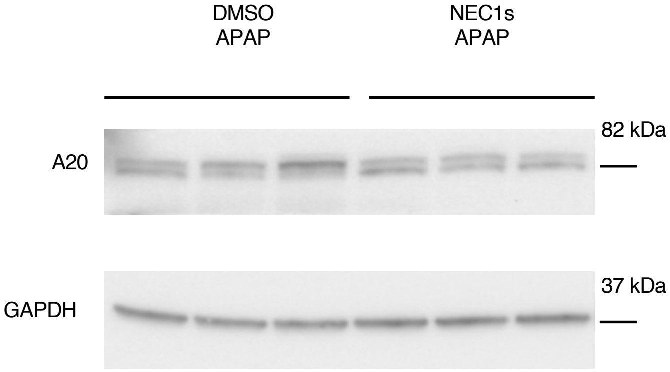

Fig. S4. Unlike RIPK1 knockdown or knockout, inhibition of RIPK1 activity with Nec1s does not affect A20 protein expression. Wild type mice were fasted overnight and treated with vehicle (DMSO) or Nec1s 45 min prior to APAP (500mg/kg) and euthanized at 24 hrs. WB of liver lysates for A20 and loading control (N=3/group). DMSO- dimethyl sulfoxide; NEC1s- necrostatin-1s; APAP-acetaminophen; GAPDH-Glyceraldehyde 3-phosphate dehydrogenase.

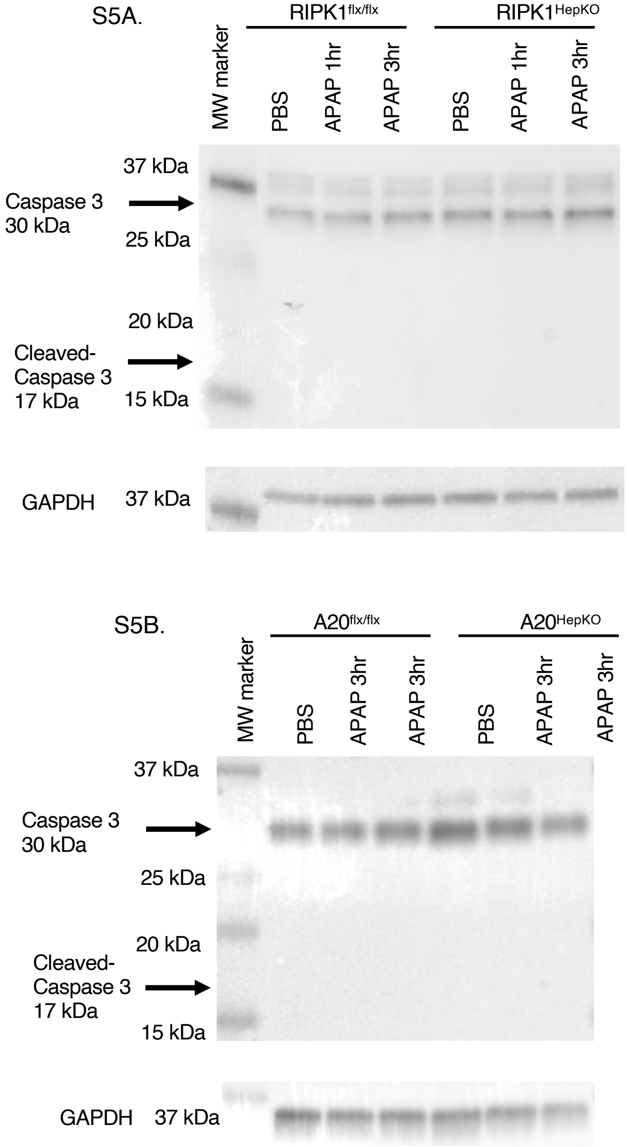

Fig. S5. No evidence of apoptosis in RIPK1^{HepCKO} and A20^{HepCKO} after APAP. (A) RIPK1$^{flx/flx}$ mice or hepatocyte specific RIPK1 knockout (RIPK1^{HepCKO}) were fasted overnight and treated with APAP (300mg/kg). WB of Caspase 3 expression and loading control. (B) A20$^{flx/flx}$ or hepatocyte specific A20 knockout (A20^{HepCKO}) were fasted overnight and treated with APAP (300mg/kg). WB of Caspase 3 expression and loading control. PBS- phosphate buffered saline; APAP-acetaminophen; RIPK1-Receptor Interacting Protein Kinase 1; GAPDH-Glyceraldehyde 3-phosphate dehydrogenase.

Fig. S6.

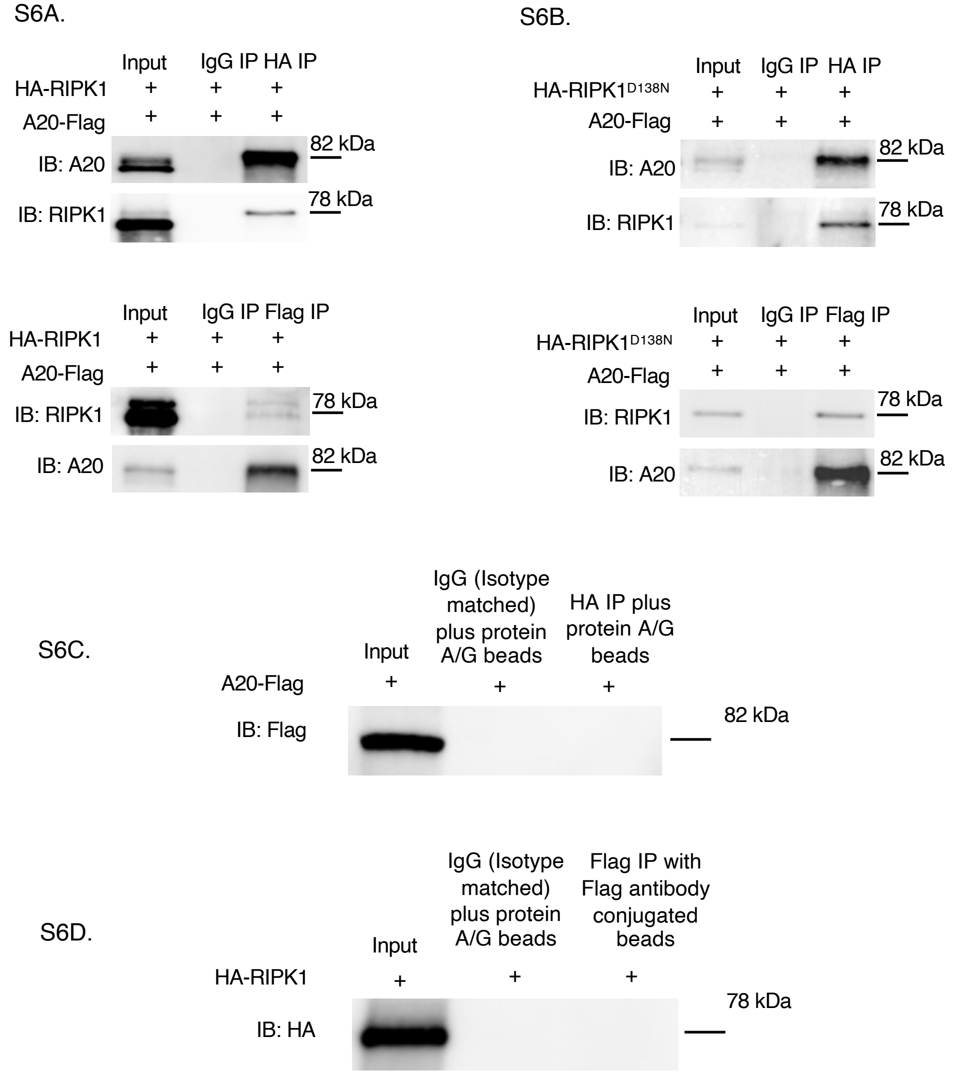

Fig. S6. A20 Co-Immunoprecipitates with RIPK1 in the hepatocyte- derived cell line, Huh7, and no non-specific binding between HA-RIPK1 and A20-Flag is detected. (A) Huh7 cells were co-transfected to overexpress HA-RIPK1 and A20-Flag for 48hrs, subsequently cells were harvested IP was performed with HA Tag, Flag Tag or isotype control IgGs. WB of A20 and RIPK1 expression in the IP samples. (B) Huh7 cells were co-transfected to overexpress the mutated HA-RIPK1-D138N and A20-Flag for 48hrs, subsequently cells were harvested IP was performed with HA Tag, Flag Tag or isotype control IgGs. WB of A20 and RIPK1 expression in the IP samples. (C) Control experiment of the HA antibody in 293 cells: A20 flag is overexpressed without HA-RIPK1 and IP is performed with either isotype-matched IgG control plus protein A/G agarose beads (middle lane) or HA antibody plus protein A/G agarose beads (right lane). WB against Flag rules out non-specific binding. (D) Control experiment for the bead-conjugated Flag antibody in 293 cells. HA-RIPK1 is overexpressed without Flag-A20 and IP is performed with either isotype-matched IgG control plus protein A/G agarose beads (middle lane) or Flag antibody-conjugated beads (right lane). WB against HA reveals no nonspecific binding.

Fig S7.

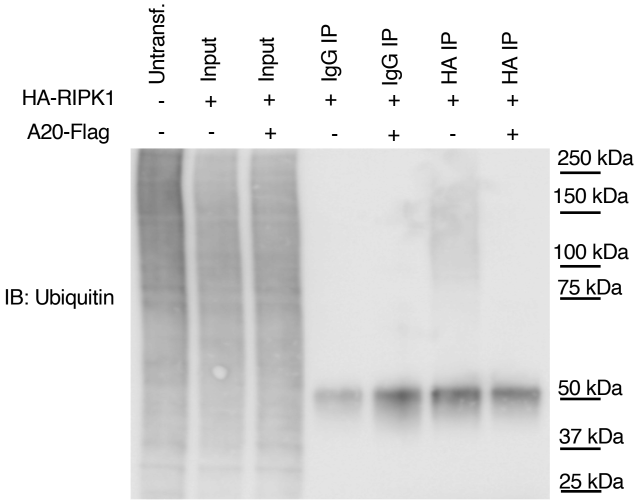

Fig. S7. RIPK1 ubiquitination status with and without A20. HEK293T cells were transfected with HA-RIPK1 in the absence and presence of A20-Flag. IP was performed 24 hrs later with HA tag and matched isotype control. WB probed against polyubiquitin.
